# Quality of life in anxious adolescents

**DOI:** 10.1186/s13034-017-0173-4

**Published:** 2017-07-20

**Authors:** Solfrid Raknes, Ståle Pallesen, Joseph A. Himle, Jon Fauskanger Bjaastad, Gro Janne Wergeland, Asle Hoffart, Kari Dyregrov, Åshild Tellefsen Håland, Bente Storm Mowatt Haugland

**Affiliations:** 1 Regional Centre for Child and Youth Mental Health and Child Welfare, Uni Research Health/University of Bergen, Bergen, Norway; 2 Department of Psychosocial Science, University of Bergen, Bergen, Norway; 3 School of Social Work, University of Michigan, Ann Arbor, USA; 4 Division of Psychiatry, Stavanger University Hospital, 4068 Stavanger, Norway; 5 Department of Child and Adolescent Psychiatry, Division of Psychiatry, Haukeland University Hospital, Bergen, Norway; 6 Research Institute, Modum Bad Psychiatric Center and Department of Psychology, University of Oslo, Oslo, Norway; 7 Faculty of Health and Social Sciences and Center for Crisis Psychology, Bergen University College, Bergen, Norway; 8 Clinic of Mental Health, Psychiatry and Addiction Treatment, Sørlandet Hospital HF, Kristiansand, Norway

**Keywords:** Anxiety, Quality of life, Adolescent at risk, Prevention, Health policy

## Abstract

**Purpose:**

To examine associations between health-related quality of life (HRQoL) and anxiety symptoms across anxiety domains (obsessions/compulsions, social anxiety, panic disorder, agoraphobia, separation anxiety, physical injury fears, generalised anxiety, and posttraumatic stress) in a general adolescent population. Expanded knowledge about these associations can provide valuable information for improving interventions and prevention strategies for adolescent anxiety.

**Methods:**

Cross-sectional data about anxiety were collected via a school survey from a community sample of Norwegian adolescents aged 12–17 (*N* = 1719). Based on scores from the Spence Children’s Anxiety Scale (SCAS), each adolescent was categorized as reporting a low, medium, or high level of anxiety. Each adolescent’s HRQoL was then measured using the Questionnaire for Measuring Health-Related Quality of Life in Children and Adolescents Revised Version (KINDL-R). Hierarchical regression analyses were performed to determine any relationship between anxiety symptoms and HRQoL.

**Results:**

Across domains of anxiety, anxiety symptoms were inversely associated with overall HRQoL. All HRQoL-dimensions were inversely associated with overall level of anxiety symptoms. In adolescents with medium and high anxiety symptoms, poor HRQoL was documented in all HRQoL dimensions with the exception of the family dimension.

**Conclusions:**

The strong association between elevated levels of anxiety symptoms and poor HRQoL demonstrate the importance of improved mental health interventions and prevention initiatives targeting anxious adolescents.

## Background

Elevated anxiety symptoms are common in youth [1, 2]. Anxiety symptoms can manifest in a broad array of situations, ranging from life-threatening situations to school presentations and sports competitions. Anxiety symptoms can enhance the individual’s ability to cope, both in dangerous situations and in situations where the individual is facing a positive, yet challenging situation [3]. In these types of situations anxiety symptoms should not be regarded as problems, but rather as resources [4]. However, anxiety symptoms are hallmarks and predictors of anxiety disorders [5]. Anxiety disorders are disabling for the individual [6, 7] and costly to society [8, 9]. Globally in 2010, anxiety disorders were among the three leading causes for disability in adolescents [10, 11]. Further, anxiety symptoms and anxiety disorders in adolescents are associated with impaired school functioning and school absenteeism, negative school environment, poor coping skills, and difficulties in relationships [6, 12, 13]. Elevated levels of anxiety symptoms during adolescence also predict subsequent depression, substance and alcohol abuse, and anxiety disorders in adulthood [5].

Preventing the onset of youth anxiety disorders is critical to avoid or at least reduce the adverse effects of anxiety on development, social functioning, and school performance. One population for whom prevention would be most beneficial is adolescents with elevated anxiety symptoms who do not yet meet diagnostic criteria for an anxiety disorder. These adolescents are regarded as having sub-threshold anxiety disorders [14] and are important to identify and intervene with in order to minimize the burden of disease [2, 10, 11]. Further, to understand and help anxious adolescents, merely summing anxiety symptoms is not enough; studies into what triggers anxiety are warranted. Consequently, the domain in which the adolescents’ anxiety appears is central to treating their anxiety problems. Specific anxiety domains (e.g. obsessions/compulsions, social anxiety, panic disorder, agoraphobia, separation anxiety, physical injury fears, generalised anxiety, and posttraumatic stress) are associated with different types of problems. An adolescent with problems in the social anxiety domain typically is troubled by situations that include social exposure, e.g. to talk with a teacher or classmates, whereas an adolescent with problems in the separation anxiety domain typically finds it difficult to be alone without anybody to talk with.

The adverse effects of an array of medical conditions on health related quality of life among adolescents (HRQoL) have been well documented [15–18]. HRQoL has been described in many different ways [19–21], with most definitions highlighting the individual’s *subjective* evaluations of life across a range of important domains [22]. Four broad health dimensions are frequently incorporated in HRQoL-definitions: physical health, mental health, social health, and functional health [22]. Improvement in any of these health dimensions is associated with increases in HRQoL, whereas worsening health is a risk factor for poorer HRQoL [23].

During adolescence, studies have reported that HRQoL decreases [24] when anxiety symptoms increase [6, 25]. In addition, female gender, ethnic minority and lower socio-economic status are factors reported as predictors of both impaired HRQoL [24, 26–29] and higher anxiety symptoms [30, 31] in adolescents. Nevertheless, only two *community*-*based* studies have investigated the associations between anxiety symptoms and HRQoL in adolescents, and both studies were based on small samples (N = 119, and N = 153, respectively). These studies reported that anxiety symptoms were negatively associated with HRQoL [32, 33]. However, neither study investigated HRQoL across specific anxiety domains (e.g. obsessions/compulsions, social phobia, panic/agoraphobia, separation anxiety, physical injury fears, and generalised anxiety). Furthermore, neither of these studies investigated *levels* of anxiety symptoms; that is, they did not separate non-anxious adolescents from adolescents with moderate and high levels of anxiety symptoms. Further, three additional studies have investigated the association between HRQoL and anxiety symptoms among adolescents with anxiety *disorders*. One of these reported that HRQoL increased as anxiety symptoms decreased in adolescents in treatment for obsessive compulsive disorder [15]. The second study reported that as parental involvement decreased and school became more demanding during adolescence, adolescents with social anxiety reported poorer HRQoL as well as higher and more impairing anxiety symptoms [34]. And interestingly, the last study found that the associations between anxiety symptoms and HRQoL were age-specific [35]. Adolescents with social anxiety often overlook positive statements from highly valued peers, feedback that could have affected them positively. This bias was neither found in socially anxious adults, nor in healthy participants. Thus, the impaired integration of social feedback, which may be an underlying mechanism that affect the relationship between anxiety and HRQoL, can be viewed as an anxiety and age specific mechanism [35].

Detailed information about the associations between various dimensions of HRQoL and anxiety domains in adolescents can be valuable for intervention improvement targeting this age group specifically.

To reduce the impact of adolescent anxiety, expanded knowledge of anxiety problems and associated problems are needed. Building on public health approaches that underscore prevention and early interventions [36–39], addressing anxiety is not only a challenge for the individual anxious adolescent, but also a challenge for schools, school health services, and policy makers. If decision makers in these systems had solid knowledge about anxiety prevention possibilities, the communities may potentially offer better prevention services, particularly important for anxious adolescents. Since the associations between anxiety symptoms and HRQoL might affect how adolescents deal with challenges such as developing a coherent and organized sense of identity, developing friendships and achieving at school [40, 41], these associations should receive attention. Knowledge about how various dimensions of HRQoL are related to anxiety symptoms could give ideas valuable for improving anxiety interventions. Furthermore, expanded knowledge about the associations between HRQoL and anxiety symptom levels can be valuable for national governments and local councils who must determine priorities for health research and make decisions about investment in health systems and interventions in the face of limited resources [39]. Public mental health prevention policies need to be based on systematic assessments of health needs [38]. Importantly, HRQoL impairment can be compared across problem types and disorders, as well as over time and communities, and are based on *subjective* information from these who experience the problem. Thus, for policy makers the HRQoL impairment associated with certain health problems can be used, together with burden of disease measurement [10, 11], to make priorities. As most anxious adolescents are struggling on their own [42], better identification and early interventions can be particularly valuable for anxious adolescents who today do not receive professional help [43].

The present study is assessing the associations between HRQoL and levels of anxiety symptoms across specific domains of anxiety within a community sample of adolescents. The aims of our study are twofold. First, we examine the degree of overall and dimension-specific HRQoL impairment in adolescents according to levels of anxiety symptoms. The assessment will add to previous research by demonstrating whether anxiety problems, indicated by level of anxiety symptoms, are associated with HRQoL in adolescents. To gain this knowledge is especially important when prioritizing between different health problems. Second, we examine the associations between HRQoL and anxiety symptoms across anxiety domains in adolescents. The knowledge gained by this investigation can be particularly valuable in the development and improvement of interventions targeting anxious adolescents.

## Methods

### Ethical approval

The study was approved by the Regional Committee for Medical and Health Research Ethics, Region West, Norway (Ethics approval No: 2013/2331 REK Vest).

### Sample and procedure

The study is based on a survey battery investigating anxiety symptoms in a community sample of adolescents aged 12–17 years. A convenience sample of ten municipalities in Norway, containing 18 lower secondary schools (grades 8–10) with a total of 4361 adolescents attending, comprised the target group. These adolescents’ parents or their other caregivers were asked to provide written informed consent for the adolescents to participate. Information about the survey was conveyed to parents through each school’s communication system and included recruitment letters via satchel mail, e-mails, SMS, and presentations by the study staff at parent meetings. The adolescents whose caregivers provided permission were invited to complete the survey in the classroom during school hours. A total of 1795 (41%) adolescents were given informed consent from caregivers to participate. Among these, 1719 adolescents (96%) completed the survey battery. Data were collected school-wise from October 2014 to June 2015. All presented data are based on the adolescents’ self-reports.

### Measures

#### Anxiety symptoms

Anxiety symptoms were assessed across several domains of anxiety and HRQoL dimensions. The Spence Children’s Anxiety Scale (SCAS) [44], child version, was used to measure adolescents’ levels of anxiety. The SCAS is a 38-item, four-point scale (range 0–114), with higher scores indicating higher levels of anxiety symptoms. The scale covers several anxiety domains including obsessions/compulsions, social phobia, panic/agoraphobia, separation anxiety, physical injury fears, and generalised anxiety. A composite anxiety score is formed by adding the subscale scores. In the present study Australian norms [45] were used, as Norwegian norms were not available. Cronbach α for the SCAC composite score was 0.92. Cronbach α for the SCAS-subscales, each comprising five to nine items, varied from 0.59 to 0.85.

#### HRQoL

The Questionnaire for Measuring Health-Related Quality of Life in Children and Adolescents Revised Version (KINDL-R) [19] was used to measure HRQoL dimensions. KINDL-R has 24 items, each answered on a five-point scale. The KINDL-R has been validated in a wide range of languages, including Norwegian [46]. The scale consists of subscales measuring dimensions of HRQoL, specifically physic (e.g. “During the last week I felt ill”), emotion (e.g. “During the past week I was bored”), self-esteem (e.g. “During the past week I felt on the top of the world”), family (e.g. “During the past week I got on well with my parents”), friends (e.g. “During the past week I got along well with my friends”) and school (e.g. “During the past week I found school interesting”). A composite HRQoL score is formed by adding the subscale scores. Mean item scores were calculated for all subscales and for the composite HRQoL scale and transformed to HRQoL subscales ranging from 0 to100. Higher scores indicated better HRQoL. For screening purposes, a composite HRQoL score of 70 is suggested as the “healthy” cut-off. Adolescents scoring above this cut-off have been identified with a moderate probability of being in good health [47]. In this study, Cronbach α for the KINDL-R total score was 0.92. Cronbach α for the KINDL-R-subscales (all with four items) varied from 0.63 to 0.87.

#### Sociodemographics and negative life events

Participants’ ethnicity was determined by asking each adolescent, “Where were you and your parents born?” Ethnicity was defined as “Norwegian” if both parents or at least one parent and the adolescent were born in Norway, “Western immigrant” if both parents or at least one parent and the adolescent were born in another Western country, and “Non-western immigrant” if both parents or at least one parent and the adolescent were born in a non-western country. Perceived family economy was assessed by the question, “How would you rate your family’s economy?” The response categories were “like most families,” “better off than most families,” and “worse off than most families.” Information about experienced negative life events was collected in the form of responses to five questions. First, a question about bullying from the Olweus questionnaire [48] was administered with a definition of bullying presented first, followed by the question, “How often have you been bullied at school in the past couple of months?” Response categories were coded on a five-point scale (1 = I have not been bullied; 2 = a few times; 3 = two or three times a month; 4 = about once a week; 5 = several times a week). Then youth were assessed regarding other negative life events by answering “Have you experienced any of the following: (1) a catastrophe or serious accident? (2) violence from an adult? (3) seen or heard anybody you care for be victim of violence from an adult? (4) unwanted sexual acts?” Responding “no” or “never” to all five questions about negative life events was coded as “0 = no negative life events” whereas responding “yes” to at least one of the items was coded “1 = reported negative life events”. These questions have preciously been used in Norwegian surveys [49].

### Anxiety level scoring

The participants were categorized as low, medium, or highly anxious, based on the overall level of anxiety symptoms indicated by the SCAS composite score. The same categorization was used separately across all anxiety domains measured by the SCAS (obsessions/compulsions, social phobia, panic/agoraphobia, separation anxiety, physical injury fears, and generalised anxiety). The term “elevated anxiety level” refers to medium and high levels of anxiety. The cut-offs were based on the Australian gender-based T-scores for adolescents. Scores that corresponded to T-scores above 65 in the Australian reference community sample were defined as high levels of anxiety symptoms; scores that corresponded to T-scores from 60 to 64 in the Australian sample were defined as medium; and scores that corresponded to T-scores below 60 in the Australian sample were defined as low levels of anxiety symptoms [45]. Hence, the cut-offs for girls on overall level of anxiety corresponded to a total SCAS-score of 38 or under = low anxiety; 39–50 = medium anxiety; 51 or above = high anxiety. And the corresponding cut offs for boys were: 32 or under = low anxiety; 33–41 = medium anxiety; 42 or above = high anxiety.

### Statistical analysis

Data were analysed using SPSS 22. Missing data varied from 2.1 to 5.9%. Due to the large sample size, skewness and kurtosis were assumed not to make a substantive impact on the analyses [50]. All participants were between 12 and 17 years old, with a majority between 13 and 15 years old. Effect sizes ‘*d*’ were calculated employing the difference of means in adolescents with low anxiety level with adolescents with medium or high anxiety levels, divided by the square of $\left( {{\text{SD}}_{1}^{2} + {\text{SD}}_{2}^{2} } \right)/ 2$ and were interpreted as small (0.20–0.50), moderate (0.51–0.80), or large (>0.80) [51]. Hierarchical multiple linear regression analyses were conducted, first to determine the contribution of levels of anxiety symptoms in predicting levels of HRQoL-total scores, and then to determine the contribution of type of anxiety on HRQoL. Each case was coded as low, medium, or high with regard to anxiety in all anxiety domains (overall, obsessions/compulsions, social phobia, panic/agoraphobia, separation anxiety, physical injury fears, and generalised anxiety). In the preliminary analyses conducted for the linear regression analyses, no violation of the assumptions of normality, linearity, and homoscedasticity appeared in the factors that were included in the analyses.

## Results

**Fig. 1:**
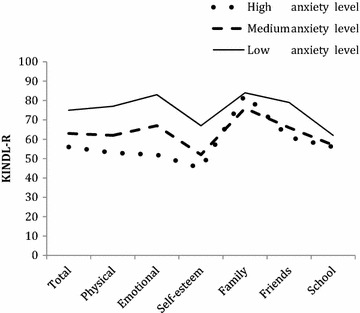
Health related quality of life (HRQoL) in all dimensions according to overall level of anxiety symptoms in a community sample of adolescents (N = 1719). The Spence Children’s Anxiety Scale (SCAS) total score was used to categorize the adolescent as high, medium, or low in anxiety symptoms. The Questionnaire for Measuring Health-Related Quality of Life in Children and Adolescents Revised Version (KINDL-R) was used to measure overall and dimension specific HRQoL

Overall HRQoL was inversely associated with levels of anxiety, as indicated by mean KINDL-R total scores of 75 (*SD* = 9.1) in adolescents with low levels of overall anxiety symptoms, 63 (*SD* = 9.2) in adolescents with medium levels of anxiety symptoms, and 56 (*SD* = 10.5) in adolescents with high levels of anxiety symptoms [F (731,630) = 13.9, *p* < 0.005]. Overall HRQoL across anxiety levels and according to socio-demographic factors, negative life events, and anxiety domains are shown in Table 1. Most adolescents in the sample were Norwegian (*n* = 1553), with a small group of Western Immigrants (*n* = 39) and Non Western Immigrants (*n* = 30). All dimensions of HRQoL were negatively associated with overall levels of anxiety symptoms, as shown in Fig. 1 and Table 2. The differences in HRQoL between adolescents with low and medium anxiety symptoms varied from small to large effect sizes ranging from *d* = 0.54 to *d* = 1.08 across HRQoL dimensions. The differences in overall HRQoL between adolescents with low and high anxiety symptoms varied from small to large effect sizes ranging from *d* = 0.14 to *d* = 2.20 across HRQoL dimensions. HRQoL in the family dimension was reported as high also in adolescents with overall elevated anxiety levels, as reflected in KINDL scores at the family dimension (mean KINDL-R family = 85; 76; 83) in adolescents with low, medium, and high levels of anxiety, respectively [F (21,651) = 76.2, *p* < 0.005).

**Table 1:** Quality of life (HRQoL) in a community sample of adolescents

	HRQoL and high anxiety level			HRQoL and medium anxiety level			HRQoL and low anxiety level		
	N	%	*M* (SD)	N	%	*M* (SD)	N	%	*M* (SD)
Overall anxiety									
Total *(N* = 1719)	115	7.1	*56.4* (10.5)	161	9.9	*62.8* (9.2)	1355	83.1	*75.4* (9.0)
Gender									
Girls	91	10.4	*55.8* (11.5)	120	13.8	*62.1* (9.1)	655	75.6	*73.3* (9.4)
Boys	24	3.4	*58.8* (9.6)	41	5.5	*64.7* (9.3)	705	91.2	*77.3* (8.2)
Age									
12–13	40	6.2	*59.7* (11.3)	55	8.6	*64.1* (9.4)	547	85.2	*78.7* (9.4)
14	39	7.1	*55.4* (10.5)	62	11.3	*62.4* (10.1)	447	81.6	*76.9* (10.7)
15–17	40	8.4	*50.8* (10.8)	47	9.8	*61.2* (8.7)	393	81.8	*74.8* (10.4)
Perceived family economy									
Normal	76	6.2	*57.5* (10.5)	113	8.9	*62.3* (8.9)	1063	84.8	*75.4* (8.9)
Higher	20	6.7	*58.8* (9.5)	33	11.4	*66.6* (9.5)	242	81.9	*76.7* (8.6)
Lower	17	21.0	*47.8* (8.7)	15	18.5	*57.8* (7.4)	48	60.5	*67.6* (10.0)
Ethnicity									
Norwegian	109	7.1	*56.2* (10.3)	149	9.5	*62.9* (9.1)	1295	80.4	*75.4* (9.0)
Western immi	3	6.8	*59.2* (7.2)	3	9.1	*62.8* (11.8)	33	84.1	*73.6* (9.7)
Non-western immi	2	9.3	*72.1* (10.0)	7	21.9	*62.3* (4.5)	21	68.8	*76.2* (8.7)
Negative life events									
No	30	3.1	*61.9* (7.9)	60	6.2	*67.1* (9.4)	883	90.7	*76.6* (8.2)
Reported	81	12.9	*54.4* (10.7)	98	15.7	*60.1* (8.0)	445	71.3	*72.8* (9.8)
Anxiety domains									
OCD	78	4.8	*58.5* (10.7)	113	6.9	*64.2* (10.8)	1440	88.2	*74.3* (9.9)
Social	142	8.7	*59.5* (11.4)	149	9.1	*65.9* (9.6)	1340	82.2	*74.9* (9.5)
Panic/agora	150	9.2	*58.1* (10.4)	214	13.1	*66.2* (9.5)	1267	77.7	*75.7* (9.1)
Separation	72	4.4	*60.1* (11.6)	125	7.7	*65.8* (10.7)	1434	87.9	*74.0* (10.2)
Physical/inj	154	9.4	*65.6* (12.6)	276	16.9	*68.4* (10.5)	1201	73.6	*74.8* (9.9)
GAD	267	16.3	*61.1* (10.7)	225	13.8	*69.4* (8.9)	1139	69.8	*76.2* (8.9)

The Questionnaire for Measuring Health-Related Quality of Life in Children and Adolescents Revised Version (KINDL-R) [19] was used to measure overall health related quality of life. The Spence Children’s Anxiety Scale (SCAS) [44], child version, was used to measure overall anxiety symptoms as well as all domains of anxiety. Australian gender-specific norms were used to categorize adolescents as low, medium, or high in anxiety symptoms. Socio-demographics are reported according to the overall anxiety level, as indicated by the SCAS total score. *Immi* immigrant. Anxiety domains: Obsessive compulsive (OCD), social phobia, panic/agoraphobia, separation anxiety, physical injury fears, and generalised anxiety (GAD)

**Table 2:** Health related quality of life in adolescents across levels of anxiety symptoms

Total HRQoL				Physical HRQoL			Emotional HRQoL			Self-esteem HRQoL			Family HRQoL			Friends HRQoL			School HRQoL		
	*M*/SD			*M*/SD			*M*/SD			*M*/SD			*M*/SD			*M*/SD			*M*/SD		
	Low	Med	High	Low	Med	High	Low	Med	High	Low	Med	High	Low	Med	High	Low	Med	High	Low	Med	High
OCD	*74.2*	*64.2*	*58.5*	*75.6*	*64.2*	*55.5*	*80.8*	*65.4*	*56.4*	*65.5*	*54.2*	*48.1*	*84.1*	*76.7*	*69.9*	*77.6*	*67.9*	*62.9*	*61.4*	*56.7*	*58.0*
	9.9	10.8	10.7	14.8	15.7	18.2	12.9	17.3	15.8	18.2	18.8	18.1	13.7	15.2	16.9	13.6	15.4	15.3	9.4	8.4	8.4
Social	*74.9*	*65.9*	*59.5*	*76.4*	*65.8*	*58.5*	*81.8*	*69.6*	*58.1*	*66.5*	*55.0*	*48.0*	*84.4*	*78.3*	*73.7*	*78.6*	*68.9*	*62.1*	*61.8*	*57.4*	*56.0*
	9.5	9.6	11.4	14.5	15.1	17.5	12.0	14.5	17.9	17.9	17.8	18.1	13.6	14.7	16.3	12.9	15.4	15.6	9.2	8.2	9.1
Panic/Agora	*75.7*	*66.2*	*58.1*	*77.6*	*64.8*	*55.1*	*82.6*	*69.9*	*56.9*	*67.3*	*56.6*	*44.8*	*85.1*	*77.5*	*71.9*	*78.8*	*69.3*	*64.6*	*61.9*	*58.6*	*55.8*
	9.1	9.5	10.4	13.8	14.3	16.3	11.4	14.9	16.4	17.5	16.9	17.6	12.9	15.2	17.7	13.0	14.3	15.9	9.2	8.8	8.6
Separation	*74.0*	*65.8*	*60.1*	*75.5*	*64.8*	*57.2*	*80.4*	*69.2*	*57.1*	*65.1*	*56.9*	*51.4*	*83.7*	*77.7*	*75.6*	*77.6*	*68.8*	*63.4*	*61.4*	*57.4*	*56.4*
	10.2	10.7	11.6	15.0	16.5	16.8	13.3	17.2	16.2	18.5	17.9	20.3	14.1	14.9	15.7	13.6	15.8	16.2	9.3	8.5	9.1
Physical/Inj	*74.8*	*68.3*	*65.6*	*76.7*	*66.9*	*64.1*	*81.0*	*72.7*	*69.9*	*65.8*	*59.7*	*55.5*	*84.4*	*79.0*	*77.9*	*77.9*	*72.6*	*69.7*	*61.9*	*59.1*	*56.4*
	9.9	10.5	12.6	14.7	15.2	18.5	13.2	15.3	18.9	18.3	18.5	19.8	13.3	15.6	17.6	13.8	14.1	16.0	9.2	8.9	9.1
GAD	*76.2*	*69.4*	*61.1*	*78.4*	*68.8*	*58.8*	*83.4*	*75.3*	*60.6*	*68.6*	*57.6*	*48.8*	*85.1*	*81.0*	*74.9*	*79.2*	*73.7*	*66.1*	*62*	*59.5*	*57.3*
	8.9	8.9	10.7	13.6	13.7	16.2	10.9	12.6	16.5	17.3	16.6	17.3	12.9	13.7	14.4	13.2	13.5	14.6	9.2	9.4	8.9
Overall anxiety	*75.4*	*62.8*	*56.4*	*77.1*	*61.8*	*52.9*	*82.5*	*66.8*	*52.4*	*66.8*	*52.4*	*44.5*	*84.8*	*75.7*	*82.9*	*78.8*	*66.0*	*60.6*	*61.8*	*56.9*	*56.2*
	9.1	9.2	10.5	13.9	14.3	16.5	11.1	17.5	15.8	17.5	17.8	17.2	13.3	14.8	14.4	12.8	14.7	15.3	9.2	8.7	8.9
Effect size		*1.37*	*1.93*		*1.08*	*1.59*		*1.07*	*2.20*		*0.82*	*1.29*		*0.65*	*0.14*		*0.93*	*1.29*		*0.54*	*0.62*
ANOVA	F(21,630) = 334.6*			F(21,654) = 223.4*			F(21,653) = 471.7*			F(21,654) = 125.3*			F(21,651) = 76.1*			F(21,636) = 157.1*			F(21,652) = 36.9*		

The Questionnaire for Measuring Health-Related Quality of Life in Children and Adolescents Revised Version (KINDL-R) [19] was used to measure health related quality of life (HRQoL). The Spence Children’s Anxiety Scale, child version, was used to measure anxiety symptoms in various anxiety domains [obsessive compulsive (OCD), social phobia, panic/agoraphobia, separation anxiety, physical injury fears, generalised anxiety (GAD), and the composite overall anxiety]. Australian gender-specific norms used to categorize adolescents as low, medium, or high in anxiety-symptom level at each domain of anxiety. The differences in overall HRQoL between adolescents with low and medium anxiety levels, and between low and high anxiety levels are reported in Cohen’s *d*. * *p* < 0.005

### Multivariate correlates of children’s mental health difficulties

Two hierarchical multiple regressions were conducted assessing correlates of HRQoL. In these regressions, gender, age, ethnicity, perceived family economic status and negative life events were entered as covariates at Step 1, followed by medium level of all anxiety domains at Step 2, and high anxiety level of all anxiety domains at Step 2 in the second analysis. Table 3 shows the results for the hierarchical regression analyses performed. Step 1 in both regression explained 23% of the variance (*R* ^2^ = 0.23). All factors except for ethnicity were significant related to the overall HRQoL, *F* (71,589) = 67.9, *p* < 0.005. Poor HRQoL was predicted by female gender, increased age, perceived low family economy, and reported negative life events. At Step 2, all domains of medium anxiety level, except generalized anxiety, held significant main effects as correlates of HRQoL. The main effects model at Step 2 accounted for 31% of the variance in HRQoL, and reached statistical significance, *F* (141,582) = 52.9, *p* < 0.005. The main and significant effects model at Step 2 in the second analysis accounted for 39% of the variance in HRQoL, *F* (131,583) = 80.2 *p* < 0.005. All domains of anxiety except separation anxiety were inversely related to quality of life in step 2 of the second analysis.

**Table 3:** Hierarchical multiple regression analyses examining effects of sociodemographic variables, reported negative life events, and levels of anxiety on quality of life in a community sample of adolescents (N = 1719)

Predictor variables	*B*	*SE B*	*β*	R^2^	ΔF
*Step 1 Background and negative life events*				*0.23* *	*67.9* *
Gender					
Female	Ref.				
Male	33.3	2.9	0.26*		
Age (12–17)	−9.9	1.7	−0.13*		
Ethnicity					
Norwegian	Ref.				
Western immigrant	2.2	9.3	0.01		
Non-Western immigrant	11.7	10.7	0.02		
Perceived family economy					
Like most families	Ref.				
Better than most families	5.1	3.8	0.03		
Lower than most families	−49.3	6.7	−0.16*		
Reported negative life events	−39.2	2.9	−0.29*		
*Step 2; Medium anxiety level (analysis 1)*				*0.31* *	*52.9* *
Gender					
Female	Ref.				
Male	28.1	5.6	0.22*		
Age (12–17)	−9.9	1.6	−0.13*		
Ethnicity					
Norwegian	Ref.				
Western immigrant	−1.5	8.8	0.00		
Non-Western immigrant	12.2	10.2	0.03		
Perceived family economy					
Like most families	Ref.				
Better than most families	6.8	3.6	0.04		
Lower than most families	−44.9	6.4	−0.15*		
Reported negative life events	−32.8	2.9	−0.25*		
Obsessive compulsive, medium anxiety symptoms	−25.7	5.6	−0.09*		
Social phobia, medium anxiety symptoms	−22.7	4.9	−0.10*		
Panic/agora fear, medium anxiety symptoms	−20.7	4.3	−0.10*		
Separation anxiety, medium anxiety symptoms	−24.8	5.3	−0.10*		
Physical injury fear, medium anxiety symptoms	−15.9	3.7	−0.09*		
Generalised anxiety, medium anxiety symptoms	−5.6	4.2	−0.03		
*Step 2; High anxiety level (analysis 2)*				*0.39* *	*80.2* *
Gender					
Female	Ref.				
Male	18.9	2.8	0.15*		
Age (12–17)	−7.6	1.5	−0.10*		
Ethnicity					
Norwegian	Ref.				
Western immigrant	−0.5	8.3	0.00		
Non-Western immigrant	14.6	9.6	0.03		
Perceived family economy					
Like most families	Ref.				
Better than most families	8.1	3.3	0.05*		
Lower than most families	−33.6	6.0	−0.11*		
Reported negative life events	−25.9	2.7	−0.19*		
Obsessive compulsive, high anxiety symptoms	−24.7	6.6	−0.08*		
Social phobia, high anxiety symptoms	−33.8	5.3	−0.14*		
Panic/agora fear, high anxiety symptoms	−35.4	5.4	−0.16*		
Separation anxiety, high anxiety symptoms	−9.9	6.9	−0.03		
Physical injury fear, high anxiety symptoms	−11.4	4.6	−0.05*		
Generalised anxiety, high anxiety symptoms	−30.4	4.5	−0.17*		

The Questionnaire for Measuring Health-Related Quality of Life in Children and Adolescents Revised Version (KINDL-R) [19] was used to measure quality of life (HRQoL). The Spence Children’s Anxiety Scale was used to measure anxiety symptoms in various anxiety domains (obsessive compulsive, social phobia, panic/agoraphobia, separation anxiety, physical injury fears, and generalised anxiety. Australian gender-specific norms used to categorize adolescents as low, medium, or high in anxiety-symptom level at each domain of anxiety. The low-anxiety group in the corresponding anxiety domain was used as the reference group in Step 2 and 3. *Ref* reference category, *β* beta estimate; * *p* < 0.005

## Discussion

This study adds to previous research by showing that both medium and high levels of anxiety symptoms are strongly associated with poor overall HRQoL. While most adolescents with low overall anxiety symptoms reported normal HRQoL, indicated by mean KINDL-R scores above the “healthy” cut-off of 70, adolescents with medium and high anxiety symptoms reported significantly poorer HRQoL. Our findings are in line with previous studies that investigated the association between HRQoL and anxiety symptoms among adolescents in community samples [32, 33] and in adolescents with anxiety disorders [15, 34, 35]. Also in line with previous studies, we found that female gender, higher age, lower perceived family economic status and reported negative life events were significantly associated with lower HRQoL and elevated levels of anxiety symptoms [24, 26]. Contrary to previous studies, ethnicity was not significantly associated with elevated anxiety symptoms or HRQoL impairment, probably due to of the small number of Non-Western immigrants participating in our sample.

Importantly, although the adolescents in our sample were recruited from a community sample and not from a help-seeking sample, elevated levels of anxiety symptoms were strongly associated with poor HRQoL. The mean level of overall HRQoL reported by adolescents with *medium* anxiety symptoms in our sample (KINDL-R total score *M* = 63) was similar to overall HRQoL reported by OCD-diagnosed, treatment-seeking Scandinavian adolescents [15]. The HRQoL impairment in adolescents with *high* overall anxiety symptoms in our sample (*M* = 56) was similar to HRQoL impairment reported by youth with bipolar disorder (*M* = 53) [16].

In health policies, the level of disability associated with a certain health problem, in this case anxiety, can be used to compare the need for investments in health services and initiatives across problem areas [23]. “Burden of disease-measurements” in developed countries report anxiety disorders as number four among the problems that cause disability [10, 11]. The results from the current study imply that adolescents with anxiety problems also report decreased HRQoL, and add to the existing literature on anxiety and HRQoL which previously primarily have been studied in adolescents with anxiety disorders. And importantly, our results add to the picture based on adolescents’ subjective reports.

Overall elevated anxiety symptom levels were associated with impairment in all HRQoL dimensions. Surprisingly however, highly anxious adolescents reported relatively high scores in the family HRQoL dimension. The finding that anxious adolescents report poor HRQoL in the school and friend dimensions and not so much in the family dimension, is in line with a previous study [15] and could have practical implications for identifying anxious adolescents. Parents are usually central in identifying problems and seeking appropriate mental health interventions for adolescents [52]. However, if the parent doesn’t witness situations in which the adolescent is impaired by anxiety symptoms, recognizing anxiety as problematic for the adolescent might be difficult. Instead, teachers are more likely to be present in these situations, suggesting that teachers are key to identifying the adolescent’s anxiety and facilitating appropriate interventions. Furthermore, since the main concern for anxious adolescents seems to be school more than family-related, early interventions targeted at anxious adolescents should consider including standard sessions for teachers and not sessions for adolescents and parents exclusively.

In health policies, the level of disability associated with a certain health problem, HRQoL impairment can be used to compare the need for investments in health services and initiatives across problem areas. “Burden of disease-measurement” in developed countries, report anxiety disorders as number four among the problems that cause disability in adolescence [11, 53]. Our findings elaborate the importance of interventions and initiatives targeting anxious adolescents on the basis of *subjective* experiences reported by adolescents themselves. Further studies should investigate the benefits and potential side effects of programs designed to increase identification and improve early interventions for anxious adolescents [54–56]. Building on public health approach underscoring preventions and early interventions [36–39], we argue that addressing anxiety is not only the responsibility of the individual adolescents and their families, but also of schools, school health services and policy makers.

### Limitations and strengths

This study has several limitations. The cross-sectional study design did not allow for conclusions about the directionality of the associations between study variables. Also the one measure point design is not ideal for conclusions about the duration of anxiety problems. Hence, the proportion of the anxious adolescents that has anxiety as a state, e.g. as a 1 day problem, is unknown. The sole use of self-report measures might also have led to a common method bias [57]. Further, family economy should be measured by other indicators than by the adolescent’s perceptions [58]. Also, in lack of Norwegian norms for the SCAS, Australian norms were applied. The mean total SCAS score for adolescent girls and boys in another Scandinavian culture (Denmark) has been found to be lower than in the Australian norm sample [59]. If the Danish norms had been used instead for Australian, more adolescents had been categorized with elevated anxiety level. Since the Danish norms were still not available on the official website for the SCAS when our analyses were run, we decided to use the Australian norms. However, for conclusions about the associations between variables and not primarily for estimating prevalence, the chosen cut-offs were not considered as a major concern. Further, the analysis was limited to anxiety problems and quality of life, and other problems that could influence the relationship between these variables were not included. There are some concerns regarding the representativeness of the study sample. Participants were recruited from three of five regions of Norway (East, South and West). The Midst and Northern regions were not included, due to practical considerations (travel distances, costs). The participating counties were situated in rural and sub-rural parts of Norway; none of them were big cities. Also, the schools were chosen on the basis that they agreed to participate in a school-based intervention study for anxious adolescents. Schools and individuals who volunteered may possess different characteristics than the population as whole, and the problem of self-selection bias may be present [60]. The reasons for the low response rate may be scepticism on the part of the parents and low priority of mental health surveys for their adolescents, in addition to technical challenges with the electronic system for collecting informed consent. Previous research has found that active parental consent lead to parental permission and response rates in the range of 30–60% for students, biased toward excluding minorities, students having problems in school, or students at risk of mental health problems [61]. However, despite these concerns, findings from our sample parallel those obtained from other epidemiological studies in adolescents attending lower secondary schools in Norway [62].

The specific strengths of the study are the high number of adolescents included and the use of well-established measures of anxiety and quality of life. The measures used in this study could be administered as screening instruments in schools to identify and help anxious adolescents. Furthermore, the study has provided knowledge on topic sparsely investigated, by documenting how medium and high levels of anxiety symptoms are associated with poor HRQoL across anxiety domains in a community sample of adolescents.

## Abbreviations

HRQoL: health related quality of life
KINDL-R: The Questionnaire for Measuring Health-Related Quality of Life in Children and Adolescents, Revised
QoL: quality of life
SCAS: The Spence Children’s Anxiety Scale
